# Cardiac T2 mapping: robustness and homogeneity of standardized in-line analysis

**DOI:** 10.1186/s12968-020-00619-x

**Published:** 2020-05-28

**Authors:** Marco Wiesmueller, Wolfgang Wuest, Rafael Heiss, Christoph Treutlein, Michael Uder, Matthias Stefan May

**Affiliations:** Department of Radiology, University Hospital Erlangen, Maximiliansplatz 3, 91054 Erlangen, Germany

**Keywords:** MRI, T2 mapping, Cardiovascular imaging, cardiovascular magnetic resonance

## Abstract

**Background and purpose:**

Interpretation of T2 values remains difficult due to limited comparability across hardware and software systems and the lack of validated measurement recommendations for the number and orientation of mandatory slices. Our aims were to provide a standardized comparison of intra- and inter-individual T2 values in the short and long axes and to investigate inter-scanner reproducibility.

**Method and materials:**

Ninety cardiovascular magnetic resonance (CMR) studies in 30 healthy subjects were performed with three identical 1.5 T CMR scanners (same hardware and software) using a balanced steady-state free precession (bSSFP) gradient echo sequence in three short axis (SAx) and three long axis (LAx) views. A commercially available T2 mapping software package of the latest generation with automatic in-line motion correction was used for acquisition. Regions of interest were manually drawn in each of the 16 myocardial segments according to the American Heart Association (AHA) model in three SAx and three LAx acquisitions. Analysis of inter-scanner, inter-segmental, intra-segmental, inter-regional and inter-level differences was performed.

**Results:**

Inter-scanner reproducibility was high and the mean myocardial T2 value for all evaluated segments was 45.7 ± 3.4 ms. Significant inter-segmental variations of mean T2 values were found. Mean intra-segmental T2 values were comparable between LAx and SAx acquisitions in 72%. Significantly higher T2 values were found in apical segments and a significant disparity among different regions was found for SAx and LAx orientations.

**Conclusion:**

Standardized cardiac T2 mapping is highly reproducible on identical CMR systems. T2 values vary significantly between single heart segments, regions, levels, and axes in young, healthy subjects.

## Background

Cardiovascular magnetic resonance (CMR) is widely used to investigate a variety of cardiac pathologies, from myocardial infarctions to non-ischemic cardiomyopathies [1–4]. Myocardial edema occurs in a variety of pathological stages, including acute myocardial infarction and inflammation. T2-weighted (T2w) CMR sequences are helpful to detect increased in vivo myocardial water content and offer a noninvasive tool to differentiate between acute and chronic disease [5]. T2w dark-blood sequences are currently used and widely accepted [6]. However, this sequence type can suffer from signal loss in higher heart rates and poor contrast between myocardium and blood in areas of insufficient blood signal suppression [7, 8]. A promising alternative technique is T2 mapping. It offers both visualization and in vivo quantification of cardiac edema and is therefore a focus of current research efforts [9, 10]. However, the highly variable intra- and inter-individual ranges of T2 values are a major drawback of and potential diagnostic pitfall for the clinical implementation of T2 values [11]. This impedes the ability to differentiate between healthy and injured myocardium, because pathologic T2 value differences can be relatively low (~ 10–20 ms) compared to this general variance [12–14]. Complex biological variations in the myocardium may serve as one explanation for these variabilities, but additional technical factors influence T2 values [15, 16]. Magnetic field strength, sequence acquisition parameters, and post-processing algorithms have been shown to limit comparability among patients at different scanner sites or in longitudinal studies [11]. Published studies address reproducibility, robustness against artifacts, and faster image acquisition protocols [17–21]. Baessler and colleagues described a robust intra-individual reproducibility of T2 values over time, whereas the inter-individual differences were comparatively high and remained unclear [15]. Recently, the Society for Cardiovascular Magnetic Resonance (SCMR) published a consensus statement to standardize the evaluation of T2 mapping based on available published evidence and expert consensus [22]. Physiologic T2 values must be established for each scanner as a benchmark to successfully implement T2 mapping in clinical routine. Unfortunately, this approach is time consuming. However, if high reproducibility could be assumed, these values could be shared among sites equipped with the same hardware and software, especially if standardization of measurement can be maintained.

The aim of this study was to provide a structured evaluation of inter-scanner reproducibility and of territorial disparity of T2 values according to the American Heart Association (AHA) model. For this purpose, T2 values in short axis (SAx) and long axis (LAx) were assessed on three identical 1.5 T CMR scanners from the same vendor, using a standardized acquisition protocol and a dedicated, commercially available software package of the latest generation with in-line motion correction and automated T2 map calculation.

## Materials and methods

### Patient population

Thirty healthy subjects were included in this prospective study. All subjects were recruited through advertising for research studies. None had a history of cardiovascular disease or cardiac risk factors, including hypertension, hyperlipidemia, or diabetes. None were referred as a patient for a clinical CMR scan. Subjects older than 30 years of age or with known cardiovascular disease, previous cardiac surgery, or relative contraindications to CMR examination (e.g., pacemaker, metal fragments, implants, arrhythmias, or claustrophobia) were excluded. Institutional Review Board approval was obtained and all patients gave written informed consent.

### T2 mapping

**Fig. 1:**
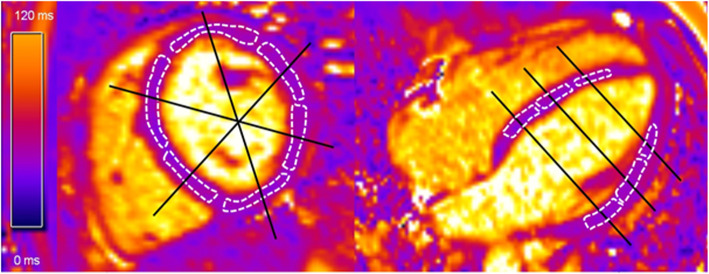
Segmental region of interist (ROI)s (dashed white lines) in basal short axis (left) and four-chamber view (right) of the same study participant. Measurement levels of short (basal, mid-cavity, and apical) and long axis (four-, three-, and two-chamber view) are referenced with black lines

All T2 measurements were performed without contrast using a balanced steady-state free precession (bSSFP) sequence (MyoMaps, Siemens Healthineers, Erlangen, Germany) on three identical 1.5 T CMR systems (MAGNETOM Aera, VE11, Siemens Healthineers). The same technologist performed all three examinations in each patient on the same day using the same 16-channel phased array body coil. The sequence of CMR scanners was randomized for each patient. Patients were placed in a supine position, and images were acquired with electrocardiogram (ECG) gating. Commands were given to hold the breath at end-expiration, as this affords high reproducibility of the position of the diaphragm. Initially, scout images were acquired to adjust the long and short axis of the heart. Three cinematic LAx (two-, three- and four-chamber view) and three SAx (basal, mid-cavity, and apical) views were acquired with a retrospectively ECG-gated segmented k-space bSSFP pulse sequence (TrueFISP, Siemens Healthineers). Basal section was defined as a fixed distance of 2 cm to the mitral annulus in an end-diastolic four-chamber view. Mid-cavity and apical sections were defined in the same four-chamber view, each with a gap of 2 cm, as shown in Fig. 1. Slice position and orientation were adopted from these acquisitions for T2 value measurements. T2 mapping parameters were as follows: Acquisition window duration, 193.27 ms; echo spacing, 2.5 ms; echo time, 1.06 ms; flip angle, 70°; preparation times, 0 ms, 25 ms, and 55 ms; field-of-view (FOV) read, 360 mm; FOV phase, 80.2%; phase resolution, 75%; slice thickness, 8 mm; base resolution, 192; voxel size, 1.87 × 1.88 × 8.0 mm^3^. Scan time was 9 heartbeats. The data were fitted using a linear two-parameter model with logarithmic transformation. Motion correction was performed using a variational non-rigid registration algorithm, aligning all T2-prepared frames to the center frame [23]. Optimal ECG gating and breath-holding were ensured and raw images as well as error maps were screened for potential image artifacts by a senior author with 8 years of experience in the field of cardiovascular imaging. In cases of suboptimal measurement, acquisition was repeated immediately.

### Map analysis

CMR analysis was performed by two board-certified radiologists (observers 1 and 2), each with 8 years of experience in cardiovascular imaging, using dedicated server/client-based post-processing software (Syngo.via VB 20, Siemens Healthineers). Both observers were blinded to all participants and other imaging data. A freehand region of interest (ROI) was manually drawn to encompass the largest possible area, carefully avoiding the adjacent blood pool or extra-cardiac structures, in each of the myocardial segments by observer 1 according to the AHA model in the three SAx (16 ROIs) and the three LAx (18 ROIs) acquisitions (Fig. 1). Heart segment 17, the apex, was excluded due to potential variability in measurements [22, 24, 25].

### Intra-observer and inter-observer evaluation

A randomly chosen subset of data from 10 subjects was measured for a second time by observer 1 for evaluation of intra-observer variability. The same subset was also measured by observer 2 for evaluation of inter-observer variability.

### Inter-scanner evaluation

T2 values from all three CMR scanners were compared intra-individually for evaluation of inter-scanner reproducibility.

### Intra-segmental evaluation

Corresponding heart segment pairs in SAx and LAx were compared to assess intra-segmental reproducibility. Segment 14 and 16 in SAx were each compared twice, first to its corresponding segment in the three-chamber view and second to its corresponding segment in the four-chamber view. This resulted in 18 corresponding matching pairs per subjects and scanner.

### Inter-segmental evaluation

For each subject, T2 values for all heart segments in SAx and LAx views were compared to each other. Pairwise post hoc tests were used to identify significant differences (e.g., segment 1 SAx to segment 2 SAx or segment 1 LAx to segment 2 LAx). In addition, inter-segmental differences were calculated for SAx and LAx together (e.g., segment 1 SAx and LAx to segment 2 SAx and LAx).

### Inter-regional evaluation

**Fig. 2:**
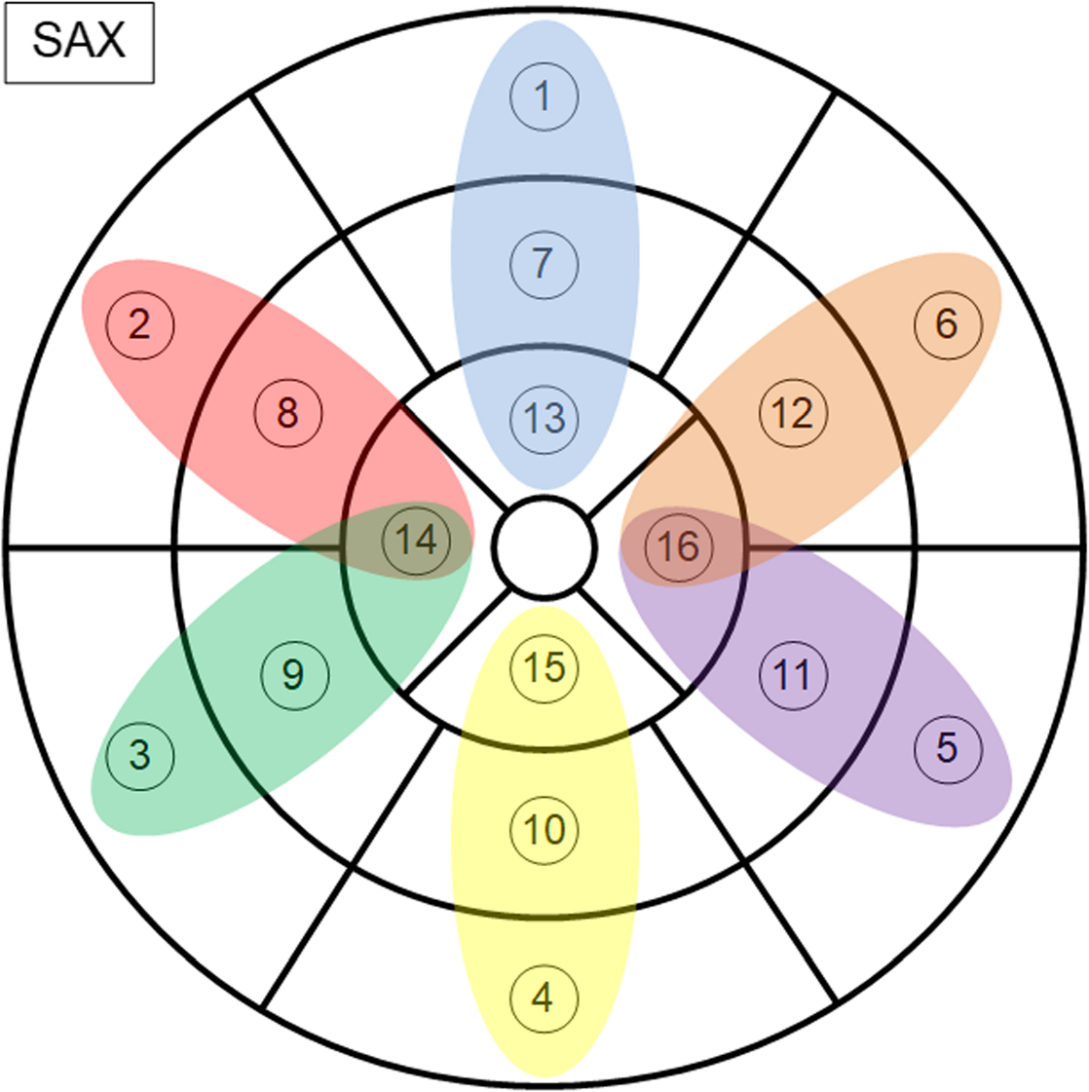
Sketch of myocardial segments in short axis (SAx) with color markings representing the groups for inter-regional analysis (anterior – blue, anteroseptal – red, inferoseptal – green, inferior – yellow, inferolateral – purple, anterolateral – orange)

To evaluate inter-regional differences, heart segments were vertically grouped into anterior (segments 1, 7, and 13), anteroseptal (segments 2, 8, and 14), inferoseptal (segments 3, 9, and 14), inferior (segments 4, 10, and 15), inferolateral (segments 5, 11, and 16), and anterolateral (segments 6, 12, and 16) regions (Fig. 2). SAx and LAx segments were grouped separately to these regions. Lateral and septal segments from the apical level (segments 14 and 16) were grouped twice to the anteroseptal/inferoseptal and anterolateral/inferolateral regions.

### Inter-level evaluation

To evaluate inter-level differences, heart segments were horizontally grouped into basal (segments 1–6), mid-cavity (segments 7–12), and apical (segments 13–16) levels. SAx and LAx segments were grouped separately to these levels.

### Subjective image quality

Both observers rated the image quality in all datasets and in each heart segment using a 3-point Likert scale: 1, insufficient image quality with major artifacts; 2, satisfactory image quality with minor artifacts; and 3, good image quality without artifacts.

### Phantom measurements

**Fig. 3:**
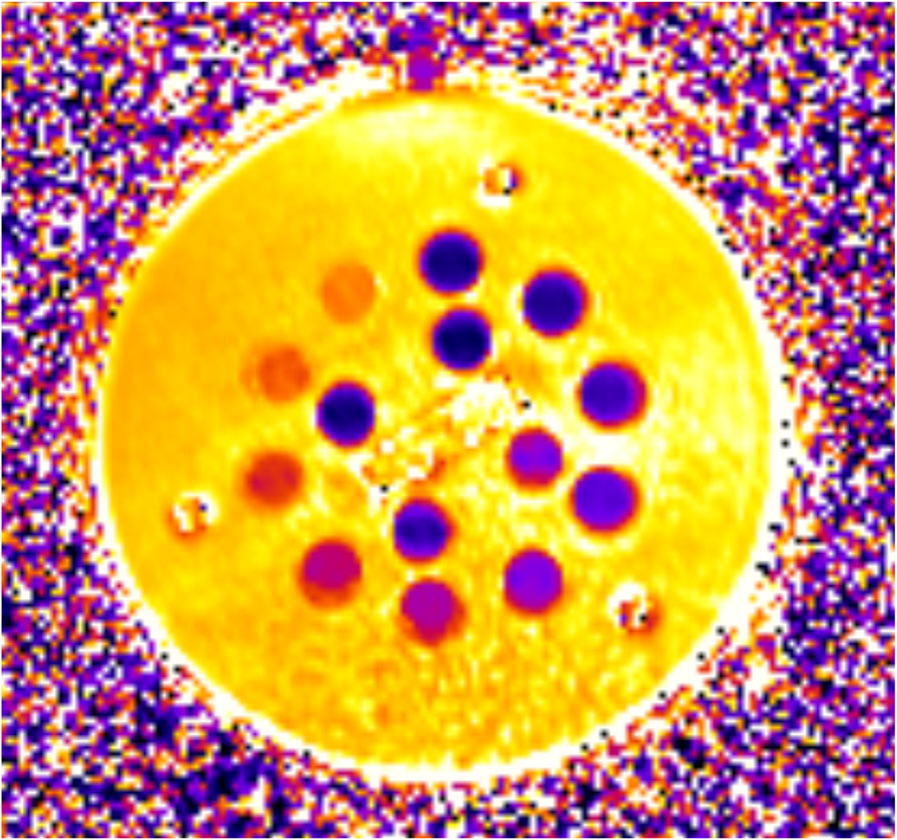
Representative image of the National Institute of Standards and Technology (NIST) phantom in axial orientation with 14 visible spheres. Darker color of the sphere indicates lower T2 values

Phantom measurements were added to the study in order to extend the evaluation of T2 mapping reproducibility to a wider range of values, including values that could be expected in pathologies, and to provide a scanner-specific reference gold standard. The XMR system phantom of the National Institute of Standards and Technology (NIST), supported by the International Society for Magnetic Resonance in Medicine (ISMRM), was chosen for these acquisitions [26]. It contains multiple layers of sphere arrays that are specifically designed for T1, T2, and proton density values. The T2 layer contains 14 different spheres with MnCl_2_-doped water (Fig. 3). We decided to measure only the 7 spheres with T2 values below 200 ms for this study because, to our knowledge, higher values are not reported in the literature for in vivo studies. Each phantom measurement was performed five times for each CMR scanner using the same dedicated bSFFP sequence with similar sequence settings as for the healthy subjects. To avoid bias, the temperature in the scan room was measured with an infrared thermometer (Bosch PTD 1, Robert Bosch GmbH, Stuttgart, Germany). Temperature differences among the three scanning rooms were below 0.5 °C (mean room temperature: 22.3 ± 0.4 °C). In addition, a one-hour waiting period between mounting the phantom and starting the measurements was observed to ensure temperature adaption of the phantom spheres.

### Statistical analysis

The Kolmogorov–Smirnov test with Lilliefors correction was used to evaluate the data for normal distribution. Descriptive statistical data were provided as mean and single standard deviation. Non-parametric Friedman test was performed for inter-scanner evaluation of T2 values among the three different CMR scanners and for inter-segmental comparison, as normal distribution was not assumed by the Kolmogorov–Smirnov test. Post hoc analysis was performed using the Dunn-Bonferroni pairwise comparison test. Bland–Altman plots were used for visual comparison of T2 values for each CMR scanner. Furthermore, the non-parametric Wilcoxon rank-sum test was used for inter-region and inter-level comparisons. Inter-observer reproducibility was assessed using the Pearson correlation coefficient for the inter-observer data in the randomly chosen datasets of 10 subjects, and intra-observer reproducibility was assessed using the intraclass correlation coefficient. The Friedman test and Pearson correlation analysis were performed for the phantom measurements. Statistical significance was accepted for *p*-values < 0.05. Statistical analysis was performed using the software package SPSS (Version 21, Statistical Package for the Social Sciences, International Business Machines, Inc., Armonk, New York, USA).

## Results

The study population consisted of 16 female and 14 male subjects with a mean age of 23 ± 4 years (range 19–29). The average heart rate during the examination was 65 ± 5 beats per minute (bpm).

**Fig. 4:**
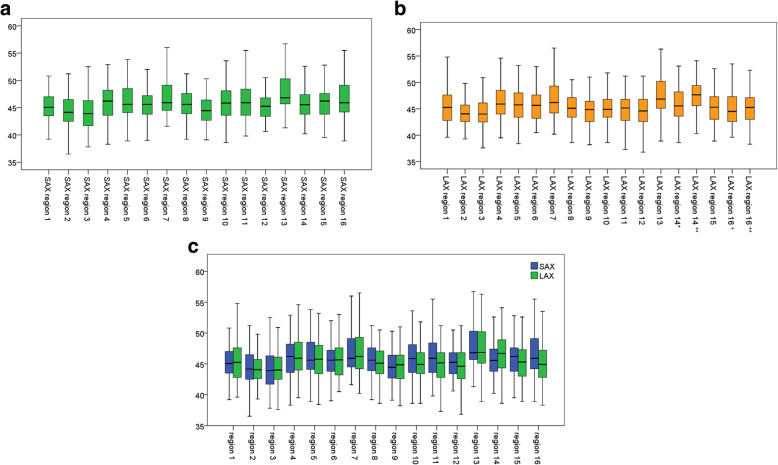
a-c Box plots of segmental T2 values in short axis (SAx), long axis (LAx), and SAx/LAx combined. Figure 4b: Single asterisks indicate apical heart regions in four-chamber view and double asterisks indicate apical heart segments in three-chamber view

A total of 102 segments per subject were analyzed: 16 segments in SAx and 18 segments in LAx at three different time points. A total of 3060 ROIs were drawn and the mean myocardial T2 value for all evaluated segments in all subjects (including all scans) was 45.7 ± 3.4 ms. Male subjects had significantly lower T2 values (44.6 ± 2.7 ms) than female subjects (46.7 ± 3.7 ms; *p* <  0.001). The mean T2 value for all segments in SAx was 45.2 ± 3.3 ms, which was significantly lower than the T2 values in LAx (46.3 ± 3.6 ms; p <  0.001). The mean intra-individual range of the T2 values (minimum value subtracted from maximum value) for all subjects was 15.7 ± 3.7 ms. The inter-individual range was 15.6 ms in a segment-based comparison. Only 5.8% of all measured T2 values were more than two standard deviations above or below the mean (6.4% in SAx and 5.2% in LAx segments). Box plots of segmental T2 values in SAx, LAx, and SAx/LAx combined are shown in Fig. 4a-c.

### Intra-observer evaluation

Intra-observer variability was low, with an intraclass correlation coefficient of 0.85.

### Inter-observer evaluation

Inter-observer variability was low, with a Pearson correlation coefficient of 0.88.

### Inter-scanner evaluation

**Fig. 5:**
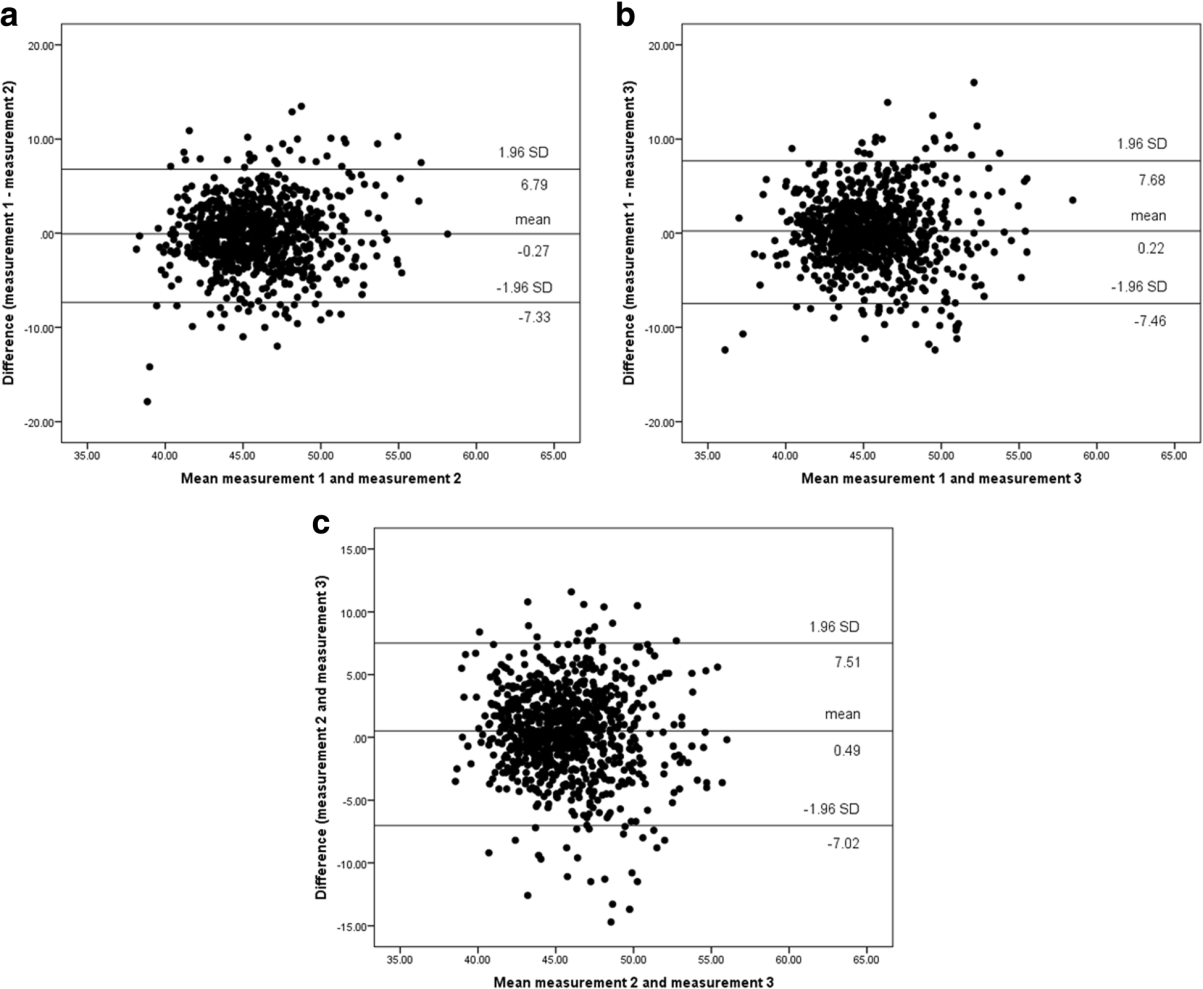
a-c Bland–Altman plots of all three time points. Horizontal lines indicate mean difference (center line) and the upper and lower border of agreement (mean difference ± 1.96 * standard deviation)

Inter-scanner variability was low, with comparable T2 values in 30 of 34 (88%) segments among the three different time points. However, segment 14 in the apical SAx (apical anterior, *p* = 0.02), segment 14 in the four-chamber LAx (apical septal, *p* = 0.01), segment 6 in the four-chamber LAx (anterolateral basal, *p* = 0.01), and segment 10 in the two-chamber LAx (inferior mid-cavity, *p* = 0.04) differed significantly among the three time points. Agreement among the different scanners is shown in Bland–Altman plots (Fig. 5 a-c).

### Intra-segmental evaluation

No differences were found in 13 of 18 (72%) corresponding myocardial segment pairs in SAx and LAx views. Segment 11 (mid-cavity inferolateral) and the apical segments 14 (septal), 15 (inferior), and 16 (lateral) differed significantly. A detailed statistical overview is provided in Table 1.

**Table 1:** Intra-segmental comparison of corresponding heart segments in short axis (SAx) and long axis (LAx). *P*-values < 0.05 are in bold print

Short Axis (SAx)	Long Axis (LAx)	*p* values
Segment 1	Segment 1 two chamber	0.815
Segment 2	Segment 2 three chamber	0.261
Segment 3	Segment 3 four chamber	0.980
Segment 4	Segment 4 two chamber	0.692
Segment 5	Segment 5 three chamber	0.389
Segment 6	Segment 6 four chamber	0.457
Segment 7	Segment 7 two chamber	0.800
Segment 8	Segment 8 three chamber	0.298
Segment 9	Segment 9 four chamber	0.903
Segment 10	Segment 10 two chamber	0.137
Segment 11	Segment 11 three chamber	**0.013**
Segment 12	Segment 12 four chamber	0.150
Segment 13	Segment 13 two chamber	0.439
Segment 14	Segment 14 four chamber	0.562
Segment 14	Segment 14 three chamber	**0.001**
Segment 15	Segment 15 two chamber	**0.020**
Segment 16	Segment 16 four chamber	**0.000**
Segment 16	Segment 16 three chamber	**0.001**

### Inter-segmental evaluation

T2 values in SAx and LAx showed significant differences (*p* <  0.001) across all segments. No discrepancies could be found in the pairwise comparisons in 77% of all segments in SAx (*p* > 0.05) and in 78% of all segments in LAx (*p* > 0.05). Inter-segment differences were slightly lower if calculated for SAx and LAx together (no significant difference in 80% of all pairwise comparisons,).

Of note, inter-segment variability of septal segments was higher than the average for both separate evaluation (50% of SAx and 60% of LAx for all pairwise comparisons with p > 0.05) and combined evaluation (64% of all pairwise comparisons with *p* > 0.05). A detailed presentation of all post hoc test results is absent due to the high number of post hoc test situations (561).

### Inter-regional evaluation

Inter-regional results were comparable in only 4 of 15 (27%) vertically grouped SAx segments and in 8 of 15 (53%) vertically grouped LAx segments. For details and descriptive data see Tables 2 and 3.

**Table 2:** Inter-regional comparison of short axis (SAx) and long axis (LAx) heart segments. Significant *p*-values are marked in bold print

Region 1	Region 2	SAx	LAx
		*p*-value	*p*-value
Anterior	Anteroseptal	**< 0.01**	**< 0.01**
Anterior	Inferoseptal	**< 0.01**	**< 0.01**
Anterior	Inferior	**< 0.01**	**< 0.01**
Anterior	Inferolateral	0.29	**< 0.01**
Anterior	Anterolateral	**< 0.01**	**< 0.01**
Anteroseptal	Inferoseptal	**< 0.01**	**< 0.01**
Anteroseptal	Inferior	**< 0.01**	0.41
Anteroseptal	Inferolateral	**< 0.01**	0.22
Anteroseptal	Anterolateral	0.07	0.15
Inferoseptal	Inferior	**< 0.01**	0.95
Inferoseptal	Inferolateral	**< 0.01**	0.60
Inferoseptal	Anterolateral	**< 0.01**	0.25
Inferior	Inferolateral	0.43	0.43
Inferior	Anterolateral	0.36	**< 0.01**
Inferolateral	Anterolateral	**< 0.01**	0.76

**Table 3:** Descriptive data for regions (anterior, anteroseptal, inferoseptal, inferior, inferolateral, anterolateral) and levels (basal, mid-cavity, apical) of heart segments in short axis (SAx) and long axis (LAx)

	Mean	Standard deviation	Range
**SAx**			
**Level**			
Basal	45.3	3.2	36.2–56.1
Mid-cavity	45.6	3.0	36.9–56.0
Apical	46.9	3.7	38.9–60.2
**Region**			
Anterior	46.7	3.5	36.9–60.2
Anteroseptal	45.4	3.1	36.2–56.6
Inferoseptal	45.0	3.2	37.8–56.6
Inferior	46.1	3.3	38.3–57.5
Inferolateral	46.4	3.5	38.9–60.1
Anterolateral	45.9	3.3	37.3–60.1
**LAx**			
**Level**			
Basal	45.3	3.4	29.9–58.0
Mid-cavity	45.3	3.3	35.6–56-5
Apical	46.2	3.7	31.9–58.4
**Region**			
Anterior	46.7	3.7	38.9–58.2
Anteroseptal	45.8	3.7	36.8–58.4
Inferoseptal	44.9	3.1	36.9–57.3
Inferior	45.5	3.5	35.6–58.0
Inferolateral	45.4	3.2	37.3–54.1
Anterolateral	45.1	3.5	29.9–56.4

### Inter-level evaluation

Basal and mid-cavity T2 values in SAx and LAx were comparable (*p* = 0.16 and *p* = 0.83, respectively). Apical T2 values in SAx and LAx were significantly higher than mid-cavity or basal values (both *p* <  0.001). A detailed overview is provided in Table 3.

### Subjective image quality

Image quality was good (Likert score = 3) in all heart segments in all CMR examinations.

### Phantom measurements

**Fig. 6:**
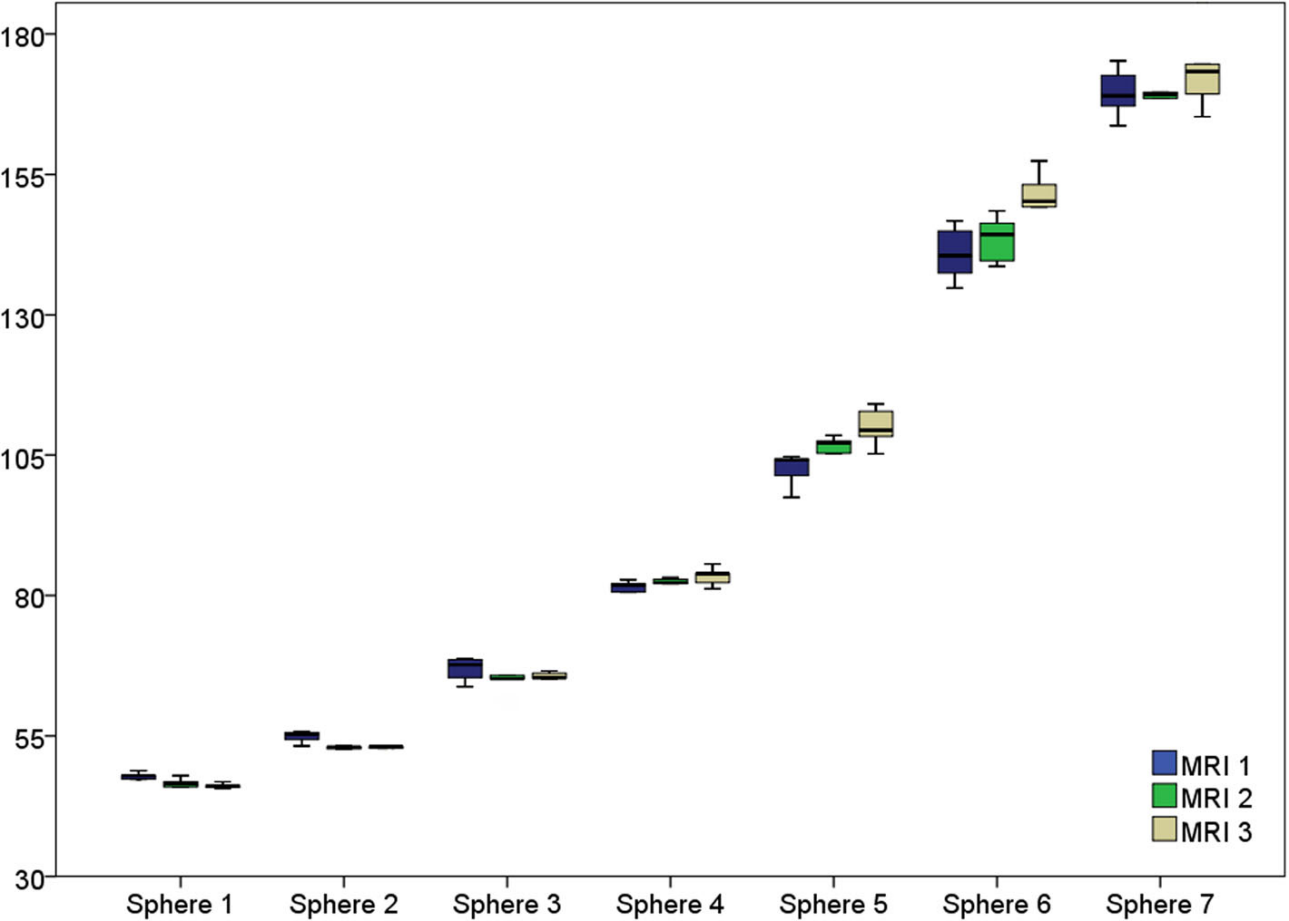
Box plots of T2 values from each phantom sphere (spheres 1 to 7) for CMR 1 to 3

No significant differences were found for the phantom measurements among the three CMR scanners (*p* = 0.347), irrespective of the height of the T2 value. The inter-scanner Pearson correlation coefficient was 0.99 for all comparison constellations. Box plots are shown in Fig. 6.

## Discussion

T2 values are highly reproducible in both the phantom and in vivo settings using identical hardware and software, as well as standardized acquisition and evaluation protocols. No significant differences were found in 88% of all assessed heart segments among three consecutive time points on three different 1.5 T CMR systems with identical hardware and software. The mean overall T2 value was 45.7 ± 3.6 ms, but comparability between corresponding SAx and LAx segments was limited, and inter-segmental, inter-regional, and inter-level differences were substantial. The accuracy is independent of the absolute height of the T2 value, as proven in a dedicated phantom.

Most of the inter-segmental differences were found in the apical segments, and these values were significantly higher than the basal and mid-cavity values. This predominantly apical variability and the significant increase of T2 values from the base to the apex in SAx and LAx is probably due to the decrease in myocardial thickness. The partial volume effect and artifacts from the surrounding air in the lungs could explain this effect and should be considered when interpreting these results. Bönner and colleagues also reported an apicobasal gradient and concluded that true morphological changes are less likely because they observed it irrespective of age and sex [24]. Mean differences between the different heart levels (basal, mid-cavity, apical) from our study were below 5 ms, which is less than the differences reported by Bönner and colleagues (5.7 ms mean difference for males and females) [24]. As these relatively small (but statistically significant) differences seem unavoidable in clinical practice, reference values should be established for all three cardiac levels if evaluation of focal changes is intended. Drawing a single ROI in the septum on mid-cavity SAx, or on basal SAx in case of artifacts, is recommended for assessment of diffuse disease and global evaluation in the consensus statement [22]. Of note, we found significant inter-segmental and inter-regional differences in the septum itself, which emphasizes the need for segmental reference values and confirms previous reports [27]. These findings are in agreement with the tight territorial distribution of different functional parameters (perfusion, metabolism, contraction) that were found by microCT analysis in a mouse model reported in the literature [28]. The variability of blood supply to septal segments 8 and 9 from the left anterior descending and the right coronary arteries could also contribute to this discrepancy. Though not completely understood, the well-established myocardium-coronary vessel interaction model may support this finding [29].

The range of inter-individual differences in T2 values from our segment-based analysis was 15.6 ms, which is in line with the literature and comparable with the mean range of intra-individual differences from this study (15.7 ms) [30]. Both inter-individual and intra-individual variation could be attributed to subject-related factors and to technical limitations [15]. However, for the majority of segments, a high inter-scanner reproducibility was obtained in our study, suggesting that the intra-individual variations, at least, truly represent individual tissue characteristics and that measurement errors are only of minor impact. Stable cut-off values for differentiating between healthy and diseased myocardium are needed. Thus far, the SCMR defines reference values within the two standard deviations from the mean [22]. Approximately 6% of all T2 values in SAx and LAx from this study were above or below this clinically normal range, although only young, healthy subjects were assessed. It remains undecided whether three standard deviations above or below the mean should be considered normal to avoid false positive findings of pathological myocardium. Unfortunately, due to our study design using only healthy subjects, we cannot yet define a new threshold or give further advice on the number or extent of pathological segments that are required for a diagnosis. However, we strongly encourage further studies to investigate this. Thus, our suggestion is to consider T2 values beyond the two-standard deviation cut-off as pathologic only when they coincide with other clinical or imaging features.

Gender also contributes to the inter-individual differences. Healthy male subjects had significantly lower T2 values than healthy female subjects. This is in line with a previous report that also found higher T2 values for young female subjects [24]. Cardiac motion was thought to be responsible for higher T2 values in females [30], but Bönner and colleagues revealed that T2 was independent of maximal systolic and diastolic strain and that differences were not attributable to different heart rates [24]. Thus, the underlying reason for higher T2 values in females remains uncertain.

In addition to individual factors, technical aspects influence the variability of T2 values. In contrast to our results, Baessler and colleagues found only moderate reproducibility of T2 values using three different acquisition techniques at three different time points on the same CMR system with subsequent manual image registration and manual motion correction [15]. We sought to exclude the potential bias from different examination techniques and from different post-processing algorithms by standardizing the acquisition technique and obtained high inter-scanner reproducibility for in vivo and phantom measurements. To our knowledge, this is the first evidence in the literature that T2 mapping can be compared across different CMR systems with the same specifications. Thus, the main challenge that remains in a setting with a standard imaging technique is subject related. Different approaches were proposed to solve this problem, ranging from texture analysis to established cut-offs for physiologic T2 values [31, 32]. In this context, we are able to provide detailed reference values for all segments in SAx and LAx for young, healthy subjects, as well as standardized phantom values for a broad range of T2 values using dedicated and commercially available software on a widely used 1.5 CMR scanner.

### Limitations

Some limitations must be considered when interpreting the results of this study. First, the number of study participants is rather small. However, the total number of CMR examinations is high, thus providing a sufficient number of replicates for robust statistical analysis. Multi-center studies with a larger number of subjects and patients could help to validate our findings.

Second, only young, healthy adult subjects were included in this study. Most patient populations are of higher age and might present with diverse medical history; thus, they might also have altered myocardial structures. Although the phantom measurements indicate that the robustness is not limited to a physiological range of T2 values, patients may be less compliant, more obese, and less capable of holding their breath than healthy subjects. However, these artifacts and problems are patient specific and should thus not be considered a technical limitation.

A third limitation is that the results are only representative for one specific type of scanner, sequence, and software. Further research is needed to transfer this information to other scanner generations and vendors. Our standardized phantom measurements could serve as starting point here. Until the proposed studies are conducted, all other scanners still require institutional reference measurements for interpretation of T2 time values, as recommended by the SCMR.

## Conclusion

Cardiac T2 mapping on identical CMR systems is highly reproducible. However, local T2 values vary significantly between single heart segments, regions, levels, and axes in young, healthy subjects. Standardized acquisition and post-processing techniques can help to address this regional disparity of the healthy myocardium during clinical interpretation and could allow for comparison among clinical sites.

## Data Availability

The datasets used and/or analyzed during the current study are available from the corresponding author on reasonable request.

## Abbreviations

AHA: American Heart Association
bSSFP: Balanced steady state free precession
CMR: Cardiovascular magnetic resonance imaging
ECG: Electrocardiogram
FOV: Field of view
ISMRM: International Society of Magnetic Resonance in Medicine
LAx: Long axis
NIST: National Institute of Standards and Technology
ROI: Region of interest
SAx: Short axis
SCMR: Society for Cardiovascular Magnetic Resonance
T2w: T2 weighted
